# Impact of Different Anthropogenic Environments on Ticks and Tick-Associated Pathogens in Alsace, a French Region Highly Endemic for Tick-Borne Diseases

**DOI:** 10.3390/microorganisms10020245

**Published:** 2022-01-23

**Authors:** Pierre H. Boyer, Cathy Barthel, Mahsa Mohseni-Zadeh, Emilie Talagrand-Reboul, Mathieu Frickert, Benoit Jaulhac, Nathalie Boulanger

**Affiliations:** 1 UR7290: Virulence Bactérienne Précoce: Groupe Borrelia, Institut de Bactériologie, FMTS, University of Strasbourg, 67000 Strasbourg, France; pierreboyer@unistra.fr (P.H.B.); cbarthel@unistra.fr (C.B.); talagrandreboul@unistra.fr (E.T.-R.); mathieu.frickert@gmail.com (M.F.); jaulhac@unistra.fr (B.J.); 2 Hôpitaux Civils de Colmar, Service de Maladies Infectieuses, 39 Avenue de la Liberté, 68000 Colmar, France; Mahsa.mohseni@ch-colmar.fr; 3 French National Reference Center for Borrelia, Centre Hospitalier Régional Universitaire, 67000 Strasbourg, France

**Keywords:** *Ixodes ricinus*, *Dermacentor reticulatus*, anthropisation, ecosystem, Lyme, *Rickettsia*

## Abstract

Ticks and tick-borne diseases have spread over the last decades. In parallel, the incidence in humans, accidental hosts for most of these zoonotic diseases, has increased. This epidemiological intensification can be associated with anthropogenic alterations of forest ecosystems and animal biodiversity, but also with socioeconomic changes. Their proliferation is largely due to human-induced effects on the factors that favor the circulation of these infectious agents. We selected different types of anthropogenic environments in Alsace, a region endemic for tick-borne diseases in France, to better understand the impact of human interventions on tick populations and tick-borne disease incidence. Ticks were collected in one golf course, three urban parks, one mid-mountain forest, and one alluvial forest that is currently part of a protected natural area. *Ixodes ricinus* was found primarily in humid vegetation, which is favorable for tick survival, such as grounds populated with trees and covered with leaf litter. We also observed that reforestation and high animal biodiversity in a protected area such as the alluvial forest led to a greater number of ticks, including both *Ixodes* *ricinus* and *Dermacentor reticulatus*, as well as to a higher prevalence of pathogens such as *Borrelia burgdorferi* sensu lato, *Anaplasma* *phagocytophilum*, *Borrelia miyamotoi*, and *Rickettsia raoulti*.

## 1. Introduction

Ticks and tick-borne diseases (TBDs) have spread since the mid-twentieth century, to a large extent due to major anthropogenic changes impacting natural ecosystems [1,2,3,4]. In the northern hemisphere, these diseases have expanded mostly due to rural desertification, land use modifications including forest culture (deforestation/afforestation) and agricultural practices, and modifications in hunting leading to an expansion in certain hosts such as deer [3,5,6]. In addition, greater awareness of clinicians and the public and improved diagnosis methods might explain the increase in TBDs [5,7]. Lyme borreliosis is the best example of diseases affected at least in part by these changes in Europe [5,6,8] and in the USA [9,10,11], constituting the first vector-borne disease of temperate regions, with the number of reported cases globally increasing [6,12,13] [Réseau Sentinelles France (sentiweb.fr), accessed on 18 January 2022].

*Ixodes ricinus* and *Dermacentor* spp. ticks are the two most widespread hard ticks in Europe [14,15]. *I. ricinus* inhabits various biotopes such as deciduous woodlands and mixed forests. Temperature and relative humidity are two key factors for its development and survival [5]. *I. ricinus* is a three-host tick with a telotropic behavior [14]. Each of its developmental stages (larvae, nymphs, and adult females) feeds once on a wide variety of vertebrate hosts including lizards, small mammals, birds, and large mammals [16]. Deer are essential to maintain the *Ixodes* population [17]. *I. ricinus* employs a questing strategy on vegetation to find its host. Nymph tick is the most important stage involved in human bite due to its small size and its wide distribution in the environment [18].

*Dermacentor* tick species, *D. marginatus* and *D. reticulatus,* are also endemic in Europe [19]. *D. reticulatus* is a three-host tick. It prefers alluvial forest and swamps, but it can also be present in drier habitats. Immature stages are endophilic and inhabit burrows of small mammals. Adults are exophilic, questing for hosts on vegetation and feeding on larger mammals such as dogs and occasionally humans [20,21]. These ticks are mainly found in a belt from the United Kingdom to Russia and from the northern part of the Iberian peninsula (Portugal and Spain) up to the southeastern coast of the Baltic Sea (Germany, Poland, and the Baltic countries) [22]. *D. marginatus* is also a three-host tick. Larvae and nymphs are endophilic ticks. Adults are exophilic and feed on larger mammals such as wild and domestic ungulates. Its main domestic host is sheep and wild animal hosts include deer, wild boar, hare, hedgehog, and occasionally humans. It inhabits more xerophilic vegetation and is present in southern Europe, with some overlap with *D. reticulatus* in central Europe [22]. The adults of both species are predominantly active in spring and autumn [5,23]. *D. reticulatus* may be locally as abundant as *Ixodes* [19].

Various microorganisms are transmitted to humans by these two tick genera: *I. ricinus* transmits bacteria (Lyme *Borrelia* spp. and *Anaplasma phagocytophilum*), parasites (*Babesia microti* and *B. divergens*), and viruses (tick-borne encephalitis virus). *Dermacentor* is known as vector of *Babesia* in dogs and horses [24,25] and *Rickettsia* including *Rickettsia slovaca* and *R. raoultii* in humans [20]. Particularly, *I. ricinus* as a generalist tick species [26] contributes to the circulation of numerous microorganisms. Small mammals such as rodents (mice, voles, dormice, squirrels, etc.) play a major role in feeding larval and nymphal ticks and constitute an excellent reservoir for *B. afzelii*, tick-borne encephalitis virus or *A. phagocytophilum* [27]. Ground feeding passerine birds, namely, the common blackbird (*Turdus merula*) and the tree pipit (*Anthus trivialis*), are also hosts for larvae and nymphs of *I. ricinus* and can be reservoirs for other *Borrelia* species such as *B. garinii* and *B. valaisiana* [28]. Large mammals like roe deer (*Capreolus capreolus*) and red deer (*Cervus elaphus*) affect the circulation of tick-borne pathogens since they can host all three tick stages [6,29,30]. These mammals are required hosts for the long and consequent bloodmeal (at least 10 days) of the larger female ticks. Although incompetent reservoirs for *Borrelia*, deer strongly influence the spreading of tick populations since they have the ability to migrate over long distances [6,12,31]. They also impact pathogen circulation, as tick co-feeding can occur on these animals allowing the transmission of pathogens from one tick to another [32]. The role of wild boar (*Sus scrofa*) on the circulation of ticks and propagation of tick-borne pathogens is less important since a more limited number of ticks feeds on them [30,33].

In Europe, the hard ticks of the genera *Ixodes* and *Dermacentor* are particularly affected by environmental modifications. Changes in human practices have modified the biodiversity, and thus, the availability of wildlife hosts to ticks [34]. To measure the impact of human practices (hunting, forest management, and urbanization) on host and tick populations, we selected different sites representing major differences in human activities: a golf course, urban parks, a mountainous site, and finally a forest destroyed by the Lothar hurricane in 1999 which was left undisturbed and unexploited since then. This major storm devastated different parts of several countries in Europe and particularly a lot of forests in France causing unprecedented damage to the ecosystems. All these sites are located in Alsace in the eastern part of France, a region with a high incidence of TBDs [35]. We selected these types of sites to describe how environmental and anthropogenic factors may modify and shape tick populations and the circulation of tick-borne pathogens. Tick activity is almost absent during wintertime in this part of France due to freezing periods and/or snow cover. Tick activity starts again in March, with a peak in April, May, and June. We selected this peak activity period to compare the density of the two tick genera, *Ixodes* and *Dermacentor*, in these different places. Furthermore, we evaluated the circulation of microorganisms, namely, *B. burgdorferi* sensu lato (s.l.) responsible for Lyme borreliosis, *A. phagocytophilum,* and *Borrelia miyamotoi* responsible for post-tick bite fever in *I. ricinus* and *Rickettsia* spp. in *D. reticulatus*.

## 2. Materials and Methods

### 2.1. Site Description

The different sites are located at various altitudes in rural, urban, and suburban areas around Strasbourg, the main city of the Rhine Valley, East of France. Altitude, longitude, and latitude were recorded with a GPS Garmin 62^®^.

Niedermunster is a forested area in the Vosges mountains at an altitude of approximately 500 m. It is a mixed forest with conifers and deciduous trees. It is regularly exploited for logs by the national forestry department. Consequently, dead trunks and branches are frequently left uncollected on the ground. The forest has an important population of deer (*Capreolus capreolus*) and boars (*Sus scrofa*) (hunters—personal communication).

The Herrenwald Forest covers an area of 77 hectares, 7 of which are used as a hunting and shooting training center. This latter forest was hardly hit by the 1999 storm where only a few large trees survived. No logging was carried out in the last 20 years to foster its transformation into a natural protected forest. Hunting training on the 7 hectares is stopped between 15 May and 31 July in order to not disturb the breeding of the European Nighthawk (*Caprimulgus europeaus)* and the European honey buzzard (*Pernis apivorus*). Boars and deer are frequent in this forest (hunters—personal communication and Inventaire National du Patrimoine Naturel (INPN), Zones Naturelles Intérêt Ecologique Floristique and Faunistique (ZNIEFF) 420030063—Forêts du Herrenwald et de Grittwald à Brumath, Vendenheim et Geudertheim—Description Museum National d’Histoire Naturelle (mnhn.fr)-2018). This biological and alluvial forest stands on a sandy terrain with layers of clay.

A golf course created in 2003 and located south of Strasbourg was also selected for this study to collect ticks. The land was fully plowed and then sowed. Mowing is stopped during the winter period and starts again in March until October, depending on the seasonal weather (7 to 8 months). The land is regularly watered (once every two days). There is no use of insecticides and acaricides. Dead leaves are left in fall and allowed to decompose. Part of the golf course is surrounded by a suburban forest where boars are present.

Ticks were also collected in three urban parks within the Strasbourg city limits. The Orangerie park was created in the XVIII century and spreads on 26 hectares. It harbors a small zoo, and some parts of the park are left without maintenance with large trees, litter layer, and shrubs with a lot of ivy. The Citadelle park was built in 1964 around the old Vauban citadel with different water plans. It spreads over 11 hectares. The Heyritz park is relatively new and covers 8 hectares along a river derived from the Ill (affluent of the Rhine), with a large population of muskrat (*Ondatra zibethicus*).

### 2.2. Field Sampling of Questing Ticks

Questing *I. ricinus* ticks were collected once every month in April, May, and June 2019 at each site. Previous tick samplings were performed in 2018 in the Strasbourg parks. Ticks were collected by dragging a 1m^2^ white flannel cloth over the vegetation. Each drag was checked after 10 m for ticks, and 30 drags were performed to cover a minimum area of 300 m^2^ as previously described [36]. For *Dermacentor* samplings, two additional collections were performed in May and June 2020 at the Herrenwald forest. Tick density was expressed per 100 m^2^.

### 2.3. Tick Identification

Tick identification was performed under a binocular loupe using the taxonomic key provided by Guglielmone et al. [20] and completed for *Dermacentor* by identification at the molecular level by sequencing the 16s rRNA gene as previously described [37].

### 2.4. DNA Extraction

Total DNA from *I. ricinus* was extracted with ammonium hydroxide 0.7M and heat as previously described [38,39]. *Dermacentor* DNA was extracted on MagNA Pure96^®^ using the MagNA Pure kit (Roche Life Sciences, Meylan, France). After grinding, total DNA was extracted according to the manufacturer’s instructions. DNA extracts were kept at −80 °C until further analyses.

### 2.5. PCR Detection of Tick-Borne Microorganisms

To assess the relative prevalence of each microorganism in ticks, for *I. ricinus* ticks a maximum of 60 ticks were individually analyzed per site and per month. For *D. reticulatus*, 70 ticks were tested for *Rickettsia* spp.

*Borrelia* detection. The presence of *B. burgdorferi* s.l. DNA in tick DNA extracts was checked by real-time PCR using primers and two Taqman^®^ probes targeting a conserved region of the *flagellin b* (Supplementary Materials, Table S1). The reactions were performed and analyzed on a LightCycler^®^480 System (Roche Life Sciences, Meylan, France).

*Borrelia* genotyping. A second real-time PCR typing assay (LightCycler^®^ 2.0 (Roche)) was performed on each positive sample in order to identify the *B. burgdorferi* s.l. species, using the same primers as those used in the first PCR assay and specific fluorescent hybridization probes (10 FRET probes and 1 TaqMan^®^ probe) as described. These probes are specific for *B. burgdorferi* s.s., *B. garinii/B. bavariensis*, *B. afzelii*, *B. valaisiana*, and *B. lusitaniae* (Supplementary Materials, Table S2). After amplification, PCR products were submitted to gradual temperature increase, and the melting temperature (Tm) of the PCR product/FRET probe duplex was analyzed. For a given species and its probe, the melting temperature (Tm) was specific.

*Anaplasma phagocytophilum* detection. *A. phagocytophilum* was detected with a real-time PCR assay targeting the *msp2*/*p44* gene on the ABI7500 PCR apparatus (Applied Biosystems, Fisher scientific, Illkirch, France) as previously described [40] (Supplementary Materials, Table S1).

Relapsing fever (RF) *Borrelia* detection and genotyping. Another real-time PCR assay was used to detect RF-*Borrelia* on a LightCycler^®^480 Instrument (Roche Life Sciences) (Supplementary Materials, Table S1) [41]. Species identification was achieved by sequencing PCR amplicons (ref https://doi.org/10.1186/s13071-020-04071-9 accessed on 7 December 2021).

*Rickettsia* detection and species identification. Detection of *Rickettsia* spp. in *Dermacentor* adults (70 tested) was done by RT-PCR assay adapted from [42] on the LightCycler^®^480 Instrument (Roche Life Sciences) targeting the *gltA* gene. To identify the *Rickettsia* species, a nested end-point PCR assay was performed targeting a 250 bp fragment of the *rompB* gene of the spotted fever group (SFG) *Rickettsia* adapted from [43]. Each amplicon was sequenced (GATC Eurofins Genomics, Basel, Switzerland) using the Sanger method with the inner primers (Supplementary Materials, Table S3).

### 2.6. Statistical Analyses

The density of nymphs (DON) is the total number of *Ixodes* ticks collected per site and per month over 100 m^2^. Nymph infection prevalence (NIP) was calculated as the number of ticks carrying a given microorganism divided by the number of tested ticks. For each collection month and site, the DIN (density of infected nymphs) is calculated as the NIP multiplied by the DON.

To compare DON, NIP, and DIN (for all microorganisms and for *Borrelia*) over the three months of collection and on the different sites, statistical modeling using generalized linear models (logistic regression for NIP and Poisson regression for DON and DIN) was performed. Data were analyzed using RStudio version 3.4.0 (2017-04-21).

## 3. Results

### 3.1. Spatiotemporal Variation of Ixodes Ricinus Nymph Density

**Figure 1:**
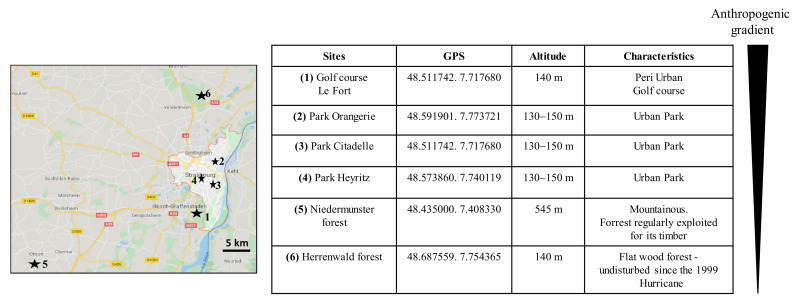
Collection sites for *Ixodes ricinus* and *Dermacentor reticulatus* in the Alsace region (France) in 2018, 2019, and 2020 and characteristics of each site. m: meters.

A total of 1,179 *I. ricinus* were collected in the selected Alsatian sites in 2018 and 2019 (Supplementary Materials, Table S4), characterized by different ecosystems and human activities (Figure 1).

The density of nymphs (DON) in 2019 varied between the sites between 0 nymphs/100 m^2^ in the Citadelle park and 56.33 nymphs/100 m^2^ in the Herrenwald forest (Supplementary Materials, Table S5). Statistical modeling (Table 1) evidenced the lowest DON on the golf course and in the three urban parks compared with Niedermunster taken as a reference. The Herrenwald forest showed the highest DON with 56.33 nymphs/100 m^2^ (RR = 1.438 *p* < 0.001). Tick activity reached its peak in all sites in May (RR_DOT-MAY_ = 1.761 *p* < 0.001) and also in parks where the tick density was comparatively low.

### 3.2. Risk of Infection

To assess the nymph infection prevalence (NIP) and the density of infected nymphs (DIN), 498 of the 1179 collected ticks were tested for *A. phagocytophilum*, RF-*Borrelia*, and *B. burgdorferi* s.l. and 73 were found positive for one microorganism (global NIP = 13.89%). No tick harbored two of these tested microorganisms. Considering all microorganisms, no statistical difference for the NIP was found between the collection sites (Table 1). However, the highest *B. burgdorferi* s.l. prevalence (NIP-*Borrelia*) was found in Herrenwald (RR_site C_ = 2.002 *p* = 0.0473). Over the three months of collection, no temporal variation of the NIP was observed, neither with all microorganisms nor with *B. burgdorferi* s.l. alone. The most prevalent microorganism was *B. burgdorferi* s.l. (NIP = 10.86%). The density of infected nymphs (DIN) was significantly higher in Herrenwald for all microorganisms (RR 1.978) as well as for *B. burgdorferi* s.l. (RR 2.719) (Table 1: DIN-*Borrelia*). No conclusion could be drawn from the analysis of the urban parks because of the low number of *Ixodes* ticks found to harbor microorganisms.

### 3.3. Repartition of the Ixodes-Borne Microorganisms According to the Site

**Figure 2:**
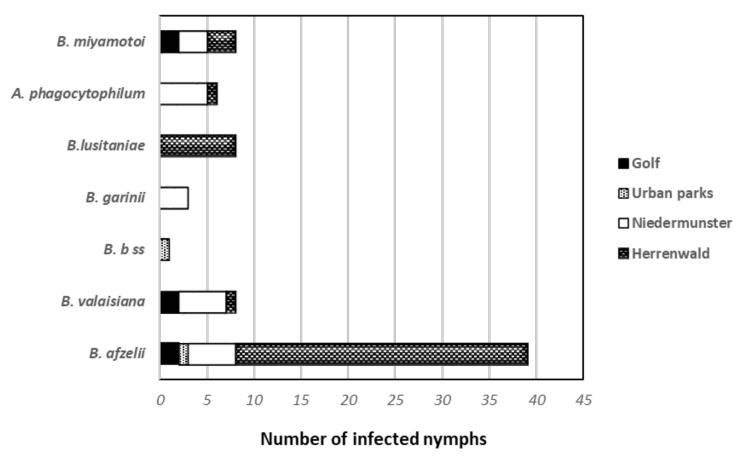
Collected *I. ricinus* nymphs were tested by PCR to detect three pathogens: *Borrelia burgdorferi* sensu lato, *Borrelia miyamotoi*, and *Anaplasma phagocytophilum*. The presence of the different microorganisms varies according to the sites: golf, urban parks, and Niedermunster and Herrenwald forests. *B. b* ss: *Borrelia burgdorferi* sensu stricto.

The two forested areas, Niedermunster and Herrenwald, presented the largest variety of microorganisms in *Ixodes* nymphs (Figure 2). The most represented genomospecies was *B. afzelii*. The most prevalent species in the Herrenwald forest after *B. afzelii* was *B. lusitaniae,* whereas *B. valaisiana* was the second most prevalent in the Niedermunster forest (Figure 2). *A. phagocytophilum* and relapsing fever (RF)-*Borrelia* were found in ticks at a level of 1.26% and 1.77%, respectively, corresponding to the level regularly found in the other Alsatian collection sites since several years [44]. Amplicon sequencing revealed that *B. miyamotoi* was the only species of RF-*Borrelia* in *I. ricinus* ticks. The two microorganisms, *A. phagocytophilum*, and *B. miyamotoi* were present in all the sites except in the park areas of Strasbourg. *A. phagocytophilum* was only detected in forested areas.

### 3.4. Dermacentor Reticulatus and Rickettsia Raoulti

A total of 275 *Dermacentor* ticks were collected (Table 2). They were all morphologically identified as *D. reticulatus*. As a confirmatory assay, a subset of 35 *Dermacentor* were submitted to 16s rRNA sequencing, with homology between 99.7% and 100% to the *D. reticulatus* gene bank sequence MK671590.1. We found an important density of this tick in the Herrenwald forest compared with the other collection sites. In March 2019, the density in this area was as high as 38 ticks/100 m^2^. In contrast, only one specimen was found in the Niedermunster forest in 2019, and no *Dermacentor* tick was found in the other sites.

Among the number of *D. reticulatus* tested for *Rickettsia* spp., 20% were positive. All *D. reticulatus* which presented a PCR positive for *Rickettsia* detection, underwent a second PCR followed by sequencing for *Rickettsia* species determination: a homology of 100% to the *R. raoultii* gene bank sequence MK304549.1 was found. No *R. slovaca* was detected.

## 4. Discussion

Ticks and TBDs have significantly expanded in our environment [11,45], particularly in Europe with the spread of *I. ricinus* up to the north [5,46,47]. The strong human impact on environment and indirect influence on the tick population dynamics are increasingly well documented. This study aimed to investigate how different anthropogenic environments with various human activities in a relatively close geographic area can affect tick density and infection risk of contracting TBDs.

We analyzed tick abundance and prevalence of three microorganisms vectored by *I. ricinus* ticks in ecosystems with strong anthropogenic pressure, such as the three urban parks of Strasbourg. The DON found on these sites was very low, indicating that the risk of being bitten by ticks is scarce if people remain on mowed grass in urban parks. Indeed, the transect in each park mainly corresponded to an environment rich in leaf litter typically found in the fringe zones or under bushes, known to provide humus and microclimatic conditions of humidity which are favorable for *I. ricinus* survival [48,49,50]. This type of environment, which is more and more promoted in urban areas to make “greener cities”, constitutes a favorable environment for *Ixodes* ticks [51]. Similarly, on the golf course, ticks were only present on the periphery of the course where vegetation is left undisturbed with significant leaf litter.

Our results join a series of works documenting that expansion of green spaces in urban areas can lead to an increase in tick populations in these zones [52]. The risk of tick exposure in urban parks in Europe was already documented in the 1990s. In the U.K., a *B. burgdorferi* infection was diagnosed in a dog after walks in a park in South London. *I. ricinus* ticks were then collected on vegetation in this park and found positive by PCR for *B. burgdorferi* DNA [53]. Urban risk was also described in 1988 in Prague [54], West Berlin [55], later in France [56], and more recently in Slovakia [57], Poland [58], and other European countries [29]. In 1997, an investigation for the presence of *I. ricinus* was conducted in the city of Lyon located south of France. Only adults were found there. The authors concluded that the tick population was unsettled [56]. In addition, it has also been documented that small rodents and medium mammals such as hedgehog [59] or fox [60], all potential hosts for any stage of *I. ricinus* ticks, populate more and more our urban and suburban areas [29]. Birds as well can easily introduce ticks in urban areas [61], predominantly bird species foraging mostly on the ground such as blackbird (*Turdus merula*) and European robin (*Erythacus rubecula*) [27]. A high connectivity between urban parks and rural areas where roe deer population (*Capreolus capreolus*) can circulate also increases the risk of tick bite and TBDs as shown by a recent study performed in Belgium [52]. Our work, together with all reports discussed above, support the conclusion that park management as well as understanding the impact of wildlife changes and ecosystem connectivity, is crucial to prevent an increase in tick population and *Borrelia*-infected ticks in urban and suburban areas.

In forested areas such as the Niedermunster and Herrenwald forests, roe deer and wild boar are known to be present in increasing density, as confirmed by hunters and forestry department staff (personal communications). Indeed, from 1973 to 2017, the population of hunted roe deer has multiplied 3.5-fold and the population of wild boar by a factor of 5.9 in France (data—Office français de la biodiversité). Ticks of the *I. ricinus* complex feed on various vertebrate species but its population is particularly maintained by roe deer (*Capreolus capreolus*) in Europe and white-tailed deer (*Odocoileus virginianus*) in North America [8,17,31,46]. Although roe deer is not a competent host for Lyme borreliosis spirochetes, it contributes to the feeding of adult and immature ticks, thus stimulating the expansion of tick populations. Deer also spread ticks long distances and favor the transmission of microorganisms by co-feeding [32]. *I. ricinus* is very sensitive to desiccation, it must therefore inhabit deciduous and mixed forests where it requires at least 80% relative humidity to survive during its off-host period [16]. The absence of forest management, such as the collection of dead wood on the ground and major accumulations of leaf litter as can be observed in the Herrenwald forest, constitutes an ideal ecotone for *I. ricinus* nymphs [62]. Both practices improve the humus layer and stimulate biodiversity. These practices are known to increase the density of ticks in the environment [50,63,64]. The obvious increase in TBDs in humans indirectly suggests that the tick population could have spread in this region [35].

Concerning the microorganisms evidenced in this study, species of the *B. burgdorferi* s.l. complex are more prevalent than other microorganisms and the species found are identical to those commonly found in this region [65]. Ticks collected in the Niedermunster forest were found to be mainly infected with *Borrelia* associated to birds (*B. garinii*), while in the Herrenwald forest a significant number of *B. lusitaniae*-infected nymphs was detected. This latter *Borrelia* species is usually rarely found in ticks in this part of Europe [66] and is mainly associated with lizards as a primary host but also with small rodents [67]. Interestingly, the prevalence of ticks carrying species of the *B. burgdorferi* s.l. complex and other tick-borne microorganisms is significantly higher in the Herrenwald forest, likely due to particular wild animals present in this protected ecosystem (420030063.pdf (mnhn.fr). In other studies, conducted in the Alsace region, additional pathogens were detected in *Ixodes* ticks such as *A. phagocytophilum* and *B. miyamotoi* and *N. mikurensis* [68,69,70,71]. In this study, *A. phagocytophilum* is the second most represented microorganism. Deer and boar populations are very good reservoirs for *A. phagocytophilum* [72,73]. We found that *Ixodes* ticks are predominantly infected with this bacterium in forested areas, rather than in urban sites. Its presence was already described in ticks collected in the south of Alsace [65] and also in other places in France [74]. However, human cases are up to now only documented in Alsace [69], suggesting the circulation of a specific ecotype [75]. The presence of *B. miyamotoi* in ticks has been reported from several regions in France [70,74,76]. However, no human case of acute disease has been documented so far in France [70], and it seems that it circulates more in forested areas than in urban parks.

For *D. reticulatus*, its range of expansion in Europe is more and more documented [15,21,23,25,77]. The high prevalence of *D. reticulatus* found in the Herrenwald forest compared with the other sites is particularly noteworthy. Its presence has so far been described mainly in the south and west of France [78,79]. It is the second most reported tick species after *I. ricinus* in Europe [19], and the patchy distribution seems to be a typical feature for its endemic pattern on the continent. Although the *D. reticulatus* presence has been described earlier in some urban city parks [80], we could not detect this tick in the visited urban parks of Strasbourg. Since the late 1970s, *D. reticulatus* has expanded all over Europe likely due to climate change and the impact of human activity on its ecosystem [11,21,81,82]. The composition of the Herrenwald forest which was left unexploited since 1999, seems to favor specific tick populations; first of all, *I. ricinus* and more particularly *Dermacentor* spp. *D. reticulatus* is mainly known as a vector of *Rickettsia slovaca* and *R. raoulti*, responsible for tick-borne Lymphadenopathy (TIBOLA) in humans [83] and *Babesia canis* and *B. caballi* in dogs and horses, respectively [25,84]. *R. slovaca* and *R. raoulti* have been diagnosed in different patients in Southern France, who were mainly bitten by *D. marginatus*. *R. slovaca* seems to be more pathogenic than *R. raoulti* [85]. In Alsace, we observed a very high incidence of *R. raoulti* in *D. reticulatus,* which accounted for 20% of all collected ticks in the Herrenwald forest. A few human cases of TIBOLA have been clinically diagnosed in Alsace which were never documented in detail, deserving further investigations (N. Boulanger—personal communication). The presence of *D. reticulatus* infected with *R. raoultii* in the east of France has been studied here for the first time. Awareness on this appearance should be raised, primarily in the clinical and diagnostic environments, since this tick and the disease are often misdiagnosed as *I. ricinus* and Lyme borreliosis, respectively.

## 5. Conclusions

Ticks and TBDs, especially the *I. ricinus* complex involved in the transmission of *B. burgdorferi* s.l. have been widespread for decades in different environments such as forests, but also more and more in parks and recreational areas [3]. In this study, although limited to three years and five sites in a restricted geographic area, we show that tick density was negatively correlated with the degree of urbanization. However, the presence of pathogens detected in our study indicates that the risk of getting a TBD in certain urban areas is not negligible, especially in fringe zones or under bushes. There is no such risk though in well-maintained areas and where the grass is cut. However, we confirm as previously described [29] that *I. ricinus* should no longer be considered as an exclusive woodland tick [86]. It inhabits urban parks in our cities [29], and also gardens in rural areas [87] when suitable microhabitats are provided. On the opposite side, abandoned wooded areas such as the Herrenwald forest constitute a highly favorable environment for the development of ticks and even more for ticks carrying potentially pathogenic microorganisms. Anthropogenic practices represent a major impact on tick populations and TBDs [3,6]. Therefore, awareness should be raised at the level of all stakeholders to reduce the quite well-known sources for favorable habitats and the expansion of ticks and TBDs in rural as well as in affected urban areas. Finally, more and larger longitudinal studies [88,89] with a multidisciplinary approach might help to better understand the importance of these anthropogenic influences.

## Figures and Tables

**Table 1:** Density of *Ixodes ricinus* nymphs (DON); nymphal infection prevalence (NIP) for all microorganisms and for *Borrelia burgdorferi* sensu lato (NIP-*Borrelia*); and density of infected nymphs (DIN) or all microorganisms and *B. burgdorferi* s.l. (DIN-*Borrelia*).

	DON: Density of Nymphs		NIP (All Microorganisms)		NIP (*Borrelia*)		DIN (All Microorganisms)		DIN (*Borrelia*)	
	Relative Risk	*p*-Value	OR	*p*-Value	OR	*p*-Value	Relative Risk	*p*-Value	Relative Risk	*p*-Value
**By Site**										
Niedermunster	Reference		Reference		Reference		Reference		Reference	
Golf	0.0515	<0.001	2.227	0.152	1.872	0.3569	0.106	0.00595	0.097	0.02556
Herrenwald	**1.438**	**<0.001**	1.454	0.225	**2.002**	**0.0473**	**1.978**	**0.02780**	**2.719**	**<0.001**
Three urban parks	0.001	0.007	0.000	0.982	0.000	0.9886	0.000	0.99693	0.000	0.99699
**By month**										
April	Reference		Reference		Reference		Reference		Reference	
May	**1.761**	**<0.001**	1.177	0.478	0.665	0.322	1.273	0.471	1.127	0.741
June	0.827	0.202	0.770	0.636	1.044	0.909	0.768	0.487	0.648	0.304

**Table 2:** Comparison of the *Dermacentor reticulatus* and *Ixodes ricinus* populations collected in the Herrenwald forest and their density per 100 m^2^. Among the *Dermacentor* ticks, 70 were tested for *Rickettsia*: 20% were positive for *R. raoulti*.

	*Dermacentor* Adult		*Ixodes* Adult		*Ixodes* Nymph	
	Total Number	Density/100 m^2^	Total Number	Density/100 m^2^	Total Number	Density/100 m^2^
March 2019	114	38	6	2	58	19
April 2019	49	16	4	1	197	65
May 2019 2020	55	18	4	1	335	111
	42	14	21	7	100	33
June 2019 2020	8	2	10	3	91	30
	7	2	17	6	84	28

## Data Availability

Available upon request.

## Supplementary Materials

The following are available online at https://www.mdpi.com/article/10.3390/microorganisms10020245/s1, Table S1: Summary of the primers and probes used for *Borrelia burgdorferi* sensu lato complex, *A. phagocytophilum* and *Borrelia miyamotoi*, Table S2: Internal fluorescently labelled probes for *B. burgdorferi* sl species detection and identification by the Melting temperature (Tm) Plus/minus standard deviation (SD)., Table S3: Summary of the primers used for *Rickettsia* identification by PCR, Table S4: (A) Total number of *Ixodes ricinus* nymphs collected on each site, (B) Number of *Ixodes ricinus* nymphs infected with different *Borrelia* species, (C) Number of *Ixodes ricinus* nymphs infected with *Borrelia miyamotoi*, (D) Number of *Ixodes ricinus* nymphs infected with *Anaplasma phagocytophilum*, Table S5: Detailed data concerning the DON, NIP and DIN of the different sites for *Ixodes ricinus.*
