# Supplementary material for: Impact of Different Anthropogenic Environments on Ticks and Tick-Associated Pathogens in Alsace, a French Region Highly Endemic for Tick-Borne Diseases
Source: Microorganisms. 2022 Jan 23;10(2):245. doi: 10.3390/microorganisms10020245 (PMC8877010; doi:10.3390/microorganisms10020245)
Supplement: Supplementary file 1 [file microorganisms-10-00245-s001.zip › microorganisms-1521401-supplementary.pdf]

Supplementary data

Table S1: Summary of the primers and probes used for *Borrelia burgdorferi* sensu lato complex, *A. phagocytophilum* and *Borrelia miyamotoi*

Pathogens	Primers (5'->3')	Probe sequence	Protocol/Reference
Borrelia burgdorferi **sensu lato complex**<br><br>**Targeted gene:**<br><br>***Flagellin - flaB***	**FlaBbsl 2a TCTTGGACITTAAGAGTTCA**<br><br>**FlaBbsl 4c GAATTAACTCCACCTTGAGIAGG**	**Mix 2/3 probe 1 and 1/3 probe 2:**<br><br>**Probe 1:** CAA GAT GAA GC [6FAM]/[TAMRA] - Sigma<br>and<br>**Probe 2:** CAA GAT GAG GC [6FAM]/[TAMRA] - Sigma	**1 cycle**: Denaturation: 95 ℃, 10 min<br><br>**55 cycles**: Hybridation / elongation: 95 ℃ - 10s then 53 ℃ – 45s<br><br>**1 cycle**: cooling 40 ℃ - 30s
Anaplasma phagocytophilum<br><br>**Targeted gene:**<br>***msp2 – p44***	**F-Anap TGTAGCTATGGAAGGCAGTGTTG**<br><br>**R-Anap GCGCTCGTAACCAATCTCAAG**	**CGGTATTGGTGGTGCCAGGGTTGA**<br>[VIC] / [TAMRA]<br>Applied biosystems/Life technologies	**(Koebel et al., 2012)**
Borrelia miyamotoi<br><br>**Targeted gene:**<br>**16S-rDNA**	**F-relfev sens GTA AAA TAC CAC RGC TCA ACT GTG with R = A and G**<br><br>**R-relfev anti-sens1 GCT TCG GTA CTA ACC TYT CGA with Y = C and T**<br>**R-relfevpersica anti-sens2**<br>**GTT AGC TTC GGT ACT AAT CTC TCA**	**TATGCTRGAAACTGCATGACTAGAGTCTGATAGG**<br><br>[6FAM]/ [BHQ1] Sigma	**(Hovius et al. 2013)**

FAM: 6-carboxyfluorescein (reporter), TAMRA: 6-carboxytetramethylrhodamine (quencher), VIC: 2′-chloro-7′phenyl-1,4-dichloro-6-carboxy-fluorescein, BHQ1: black hole quencher1 (quencher). LNA® (Locked nucleic acid) bases are underlined.

Table S2. Internal fluorescently labelled probes for *B. burgdorferi* sl species detection and identification by the Melting temperature (Tm) Plus/minus standard deviation (SD).

Probe	Technology used	Species specificity	Nucleotide sequence from 5' to 3'	Fluorescent dyes	Nucleotide location from initiation codon of B31 strain	Tm +/- SD Melting temperature
FlaBbss 3'Fluo<br>*FlaBbss* 5'LC Red	LightCycler® Probes	*B. burgdorferi* sensu stricto	GAG GGA GCT CAA ACT GCT CAG GCT GC<br>CGG TTC AAG AGG GTG TTC AAC AGG	3' fluorescein<br>5' LC Red 705	784 - 809<br>812 - 835	67.4 +/- 0.3
FlaBg 3'Fluo<br>*FlaBg* 5'LC Red	LightCycler® Probes	*B. garinii/B. bavariensis*	AGC AAA TCA AGA TGA (inosine)GC GAT TGC TGT<br>ATA TTT ATG CTG C(inosine)A ATG TTG CAA ATC T	3' fluorescein<br>5' LC Red 640	717- 743<br>746- 773	60.0 +/- 0.2
FlaBaf 3'Fluo<br>*FlaBaf* 5'LC Red	LightCycler® Probes	*B. afzelii*	AAA TCT TTT TGC TGG TGA GGG AGC T<br>AAG CTG CTC AGG CTG CAC CTG TTC A	3' fluorescein<br>5' LC Red 705	768 - 792<br>794 - 818	65.1 +/- 0.4
FlaBval 3'Fluo<br>*FlaBval* 5'LC Red	LightCycler® Probes	*B. valaisiana*	AAT CTG TTT TCT GGT G<br>AGC TCA A(inosine)C TGC TC	3' fluorescein<br>5' LC Red 640	769 - 784<br>789- 802	55.0 +/- 0.4
FlaBlus 3'Fluo<br>*FlaBlus* 5'LC Red	LightCycler® Probes	*B. lusitaniae*	AGA CTG CAC CTG CTC AAG AAG GTG TT<br>AAC AAG AAG GAG CTC AAC AAC CAG CGC	3' fluorescein<br>5' LC Red 705	804 - 828<br>830 - 856	67.3 +/- 0.4

TAMRA, 6-carboxytetramethylrhodamine (quencher). FAM: 6-carboxyfluorescein (reporter). LNA® (Locked nucleic acid) bases are underlined.

Table S3: Summary of the primers used for *Rickettsia* identification by PCR

Pathogens	Primer/Nucleotide sequence (5'->3')	Probe sequence	Protocols	References
Rickettsia* spp**<br><br>**Targeted gene:**<br>gltA***	**Rspp-F :**<br>**GAGAGAAAATTATATCCAAATGTTGAT**<br><br>**Rspp-R : AGGGTCTTCGTGCATTTCTT**	**CATTGTGCCATCCAGCCTAC GGT**<br>[6FAM]/ [BHQ1] Sigma	**1 cycle:** Denaturation: 50 ℃- 2min and 95 ℃- 10min<br><br>**50 cycles:**Hybridation/elongation: 95 ℃- 15sec and 60 ℃- 1min<br><br>**1cycle**: cooling 40 ℃-30sec	**(Labruna et al., 2004)**
Rickettsia* spp.**<br><br>**Targeted gene :**<br>rompB***	**First PCR**<br><br>**Rc.rompB.4362p:**<br>**GTCAGCGTTACTTCTTCGATGC**<br>**Rc.rompB.4,836n:**<br>**CCGTACTCCATCTTAGCATCAG**		**1 cycle**: Denaturation : 98 ℃- 30sec<br><br>**35 cycles** : Denaturation :98 ℃-15sec<br>Hybridation : 54 ℃-15sec<br>Elongation: 72 ℃ - 30s<br><br>**1cycle** :Elongation: 72 ℃-5min +4 ℃ :∞	**(Choi et al., 2005)**
	**Nested PCR**<br><br>**Rc.rompB.4,496p:**<br>**CCAATGGCAGGACTTAGCTACT**<br>**Rc.rompB.4,762n:**<br>**AGGCTGGCTGATACACGGAGTAA**		**1 cycle** : Denaturation: 98 ℃- 30sec<br><br>**35 cycles** :Denaturation: 98 ℃-15sec<br>Hybridation: 56 ℃-15sec<br>Elongation: 72 ℃ - 30s<br><br>**1cycle**: Elongation: 72 ℃-5min +4 ℃ :∞	**(Choi et al., 2005)**

Table S4

(A) Total number of *Ixodes ricinus* nymphs collected on each site

Site	**Golf**	**Citadelle**		**Heyritz**		**Orangerie**		**Niedermunster**	**Herrenwald**
Year	2019	2018	2019	2018	2019	2018	2019	2019	2019
March	ND	ND	ND	ND	ND	ND	ND	14	58
April	0	3	0	0	2	9	12	99	197
May	13	1	0	2	0	11	0	183	326
June	9	1	0	0	0	3	0	145	91

(B) Number of *Ixodes ricinus* nymphs infected with different *Borrelia* species

Site	**Golf**	**Citadelle**		**Heyritz**		**Orangerie**		**Niedermunster**	**Herrenwald**
Year	2019	2018	2019	2018	2019	2018	2019	2019	2019
March	ND	ND	ND	ND	ND	ND	ND	1	15 (13 Baf + 2Blusit)
April	0	1 (Bval)	0	0	0	2 (Bbss+Baf)	0	6 (3 Bgar + 3 Bval)	10 (8 baf + 2 Blusit)
May	1 (Bval)	0	0	0	0	0	0	2 (Baf + Bval)	7 (5 Baf + 2 Blusit)
June	2 (Baf)	0	0	0	0	0	0	5 (4 Baf + 1 Bval)	8 (5 Baf + 2 Blusit + 1 Bval)

(C) Number of *Ixodes ricinus* nymphs infected with *Borrelia miyamotoi*

Site	**Golf**	**Citadelle**		**Heyritz**		**Orangerie**		**Niedermunster**	**Herrenwald**
Year	2019	2018	2019	2018	2019	2018	2019	2019	2019
March	ND	ND	ND	ND	ND	ND	ND	0	1
April	0	0	0	0	0	0	0	2	0
May	1	0	0	0	0	0	0	1	1
June	1	0	0	0	0	0	0	0	1

(D) Number of *Ixodes ricinus* nymphs infected with *Anaplasma phagocytophilum*

Site	**Golf**	**Citadelle**		**Heyritz**		**Orangerie**		**Niedermunster**	**Herrenwald**
Year	2019	2018	2019	2018	2019	2018	2019	2019	2019
March	ND	ND	ND	ND	ND	ND	ND	1	0
April	0	0	0	0	0	0	0	1	0
May	0	0	0	0	0	0	0	1	0
June	0	0	0	0	0	0	0	2	1

Table S5: Detailed data concerning the DON, NIP and DIN of the different sites for *Ixodes ricinus*

Site	Density in *Ixodes* nymph (DON) / 100 m^2 [CI 95 %]		Nymphal infection prevalence (NIP) Whatever the microorganism [CI 95 %]		Density in *Ixodes* infected nymph (DIN) /100 m^2 whatever the microorganism [CI 95 %]	
	**2018**	**2019**	**2018**	**2019**	**2018**	**2019**
**Golf**	-	**2.44** [-3.07-7.96]	-	**0.16** [-0.25-0.58]	-	**0.56** [-0.7093-1.82]
**Citadelle**	**0.56** [-0.40-1.51]	0	**0.11** [-0.37-0.59]	0	0.11 [-0.38-0.59]	0
**Heyritz**	**0.22** [-0.73-1.18]	**0.22** [-0.73-1.18]	0	0	0	0
**Orangerie**	**2.56** [-0.90-6.00]	**1.33** [-4.4-7.10]	**0.07** [-0.24-0.39]	0	0.22 [-0.73-1.18]	0
**Niedermunster**	-	**36.75** [-1.85-75.35]	-	**0.08** [-0.008-0.160]	-	**1.83** [0.18-3.48]
**Herrenwald**	-	**56.33** [-10.67;123.3]	-	**0.12** [-0.08-0.32]	-	**3.67** [1.829-5.504]
